# Salivary gland cancer in Southern Brazil: a prognostic study of 107 cases

**DOI:** 10.4317/medoral.24248

**Published:** 2020-11-28

**Authors:** Virgilio Gonzales Zanella, Vivian Petersen Wagner, Tuany Rafaeli Schmidt, Stefanie Thieme, Cintia Correa, Felipe Paiva Fonseca, Pettala Rigon, Marinez Bizarro Barra, Ricardo Gallicchio Kroef, Pablo Agustin Vargas, Manoela Domingues Martins

**Affiliations:** 1 Head and Neck Surgery Department, Santa Rita Hospital, Santa Casa de Misericórdia de Porto Alegre, Porto Alegre, RS, Brazil; 2 Department of Pathology, School of Dentistry, Federal University of Rio Grande do Sul, Porto Alegre, Brazil; 3 Department of Oral Diagnosis, Piracicaba Dental School, University of Campinas, Piracicaba, Brazil; 4 Department of Oral Surgery and Pathology, School of Dentistry, Universidade Federal de Minas Gerais, Belo Horizonte, Brazil.; 5 Pathology Service, Santa Rita Hospital, Santa Casa de Misericórdia de Porto Alegre, Porto Alegre, RS, Brazil; 6 Department of Oral Biology and Pathology, School of Dentistry, University of Pretoria, Pretoria, South Africa

## Abstract

**Background:**

Salivary gland cancers (SGC) represent an uncommon group of heterogeneous tumors. We performed a retrospective survey of SGC diagnosed in a reference center for treatment of malignant tumors from the south of Brazil aiming to determine the prognostic value of demographic, clinic and pathologic features.

**Material and Methods:**

Cases diagnosed as SGC between 2006 and 2016 were retrospectively collected. Medical records were examined to extract demographic, clinic, pathologic and follow-up information.

**Results:**

One-hundred and seven cases of SGC were identified. The most common SGC were mucoepidermoid carcinoma (MEC) (n = 39) followed by adenoid cystic carcinoma (AdCC) (n = 29). Among AdCCs, 55.2% of cases were classified as cribriform, 27.6% as tubular and 17.2% as solid. The tubular subtype had the highest percentage of cases with perineural invasion (*p*=0.01). Among MEC, 61.5% of cases were classified as low grade, 15.4% as intermediate grade and 19.9% as high grade. Low grade MEC had the lowest percentage of cases with perineural invasion (*p*=0.04). The 5-year survival for loco-regional control, disease-free survival (DFS) and disease-specific survival were 75%, 70% and 84%, respectively. The following features were associated with poor DFS: advanced age (*p*=0.03), rural residency (*p*=0.01), being a smoker or former smoker (*p*=0.01), pain (*p*=0.03), nodal metastasis (*p* <0.001), need for chemotherapy (*p*=0.02), neck dissection (*p*=0.04), perineural invasion (*p*=0.01), and being diagnosed with AdCC compared to MEC (*p*=0.02).

**Conclusions:**

The clinco-demographic and pathologic features identified as prognostic factors reveal the profile of patients at increased risk of recurrence and who would benefit from closer follow-up.

** Key words:**Head and neck neoplasms, neoplasms, glandular and epithelial, rare diseases, epidemiology, follow up studies.

## Introduction

In 2018, IARC estimated 52,799 new cases of salivary gland cancers (SGCs) worldwide (1). This number is fairly modest compared to highly prevalent tumors such as lung and breast cancers. SGCs are unusual and, therefore, face many challenges associated with rare cancers such as late and incorrect diagnosis, limited clinical expertise, and minimal research interest (2). It is important to highlight that the IARC also estimates an increase of more than 55% in new SGC cases between 2018 and 2040, reaching an annual global incidence of 82,039 by that year. This escalation does not change the fact that SGCs represent a rare group of tumors; however, this increase will result in more surgeons and oncologists having to deal with SGC cases in the next decades. Inexperienced or less experienced professionals will need to update their knowledge concerning SGCs based on reliable scientific evidence to provide accurate diagnosis, management, and follow-up.

SGCs are also acknowledged as a microscopically diverse group of human neoplasms (3). The latest classification proposed by the World Health Organization (WHO) recognizes more than 20 types of SGC, representing a major challenge to both pathologists and clinicians as a result of an enormously heterogeneous microscopic appearance combined with mixed clinical behavior (4). Data from the literature suggest that the prevalence of SGC types varies between each geographic region. Whereas the majority of studies determined that mucoepidermoid carcinoma (MEC) is the most prevalent SGC (5,6), some authors from Turkey and Croatia have found that adenoid cystic carcinoma (AdCC) is more common (7,8), with a previous study from the south of Brazil corroborating with these latest data (9).

Epidemiological surveys are an important tool to better understand how a disease behaves within a specific population by evaluating its main demographic, clinical, and pathologic characteristics, and how such features can influence the outcomes during follow-up. For rare cancers, more specifically, retrospective surveys might represent an effective instrument to determine aetiologic and prognostic factors leading to a better comprehension of populations that are at an increased risk of developing the disease or those who might benefit from adjuvant treatments or closer follow-up (2). There is a lack of recent surveys with a representative sample of SGC in southern Brazil. Moreover, most of the studies conducted with Brazilian populations fail to evaluate prognostic factors in SGC due to limited information on follow-up. Therefore, the aim of the present study was to perform a retrospective survey of all SGC diagnosed in an 11-year period at a major reference center for malignant tumors in the south of Brazil and to determine the prognostic value of demographic, clinical, and pathological features based on a representative period of follow-up.

## Material and Methods

- Study design and patients

All patients diagnosed with SGC between January 2006 and December 2016 at Santa Rita Hospital - Irmandade da Santa Casa de Misericordia de Porto Alegre were identified. Search criteria were made on the basis of ICD-10 coding as well as on combinations of topography and morphology codes at our Pathology Service. The medical records were manually evaluated to recover information about sociodemographic characteristics (gender, age, skin color, residency), type of healthcare system (public, health insurance, or private), smoking habit, clinical features [site, clinical aspects, pain, paresthesia, size, and clinical stage based on the American Joint Committee on Cancer (AJCC) 8th edition (10)], treatment, and follow-up information (presence of recurrence, metastasis, or death). Date of diagnosis, recurrence, metastasis, death (when available), and last follow-up were noted for survival analysis. Final histopathological diagnosis and other histopathological features (grade, microscopic growth pattern, perineural and perivascular invasions) were retrieved from the Pathology Service Report.

Slides stained with hematoxylin and eosin of all cases were reviewed by two experienced head and neck pathologists. Final diagnoses were established based on the latest WHO criteria (4). When necessary, special stains, such as periodic acid-Schiff or mucicarmine, and immunohistochemical markers were performed to confirm the diagnoses. For all cases, perineural and perivascular invasions were assessed. Cases diagnosed as adenoid cystic carcinoma (AdCC) were classified according to the histopathological pattern in cribriform, tubular, or solid. Cases diagnosed as mucoepidermoid carcinoma (MEC) were graded according to the criteria proposed by the Armed Forces Institute of Pathology – AFIP in low, intermediate, and high grade (11).

- Statistical Analysis

Descriptive statistics were reported for patient and disease characteristics. The association with clinical and histological features was analyzed by a Chi-square test for categorical covariates. Differences in numerical covariates were assessed through an ANOVA test followed by a Tukey post-hoc test (for parametric data) or Kruskal-Wallis test followed by a Tukey post-hoc test (for non-parametric data). Locoregional control (LRC), disease-free survival (DFS), and disease-specific survival (DSS) were established, respectively, based on differences between date of diagnosis and date of locoregional recurrence, locoregional recurrence or late distant metastasis, and disease-associated mortality. Other outcomes, such as mortality related to other causes, were considered as censored events. The Kaplan-Meier method was used to produce survival estimates of LRC, DFS, and DSS. The 5-year event-free rate and its standard error were extracted from the Kaplan-Meier analysis. Univariate survival analysis was carried out with a Cox proportional hazards model. Survival curves were constructed for covariates significantly associated with DFS and compared using the log-rank test. All analyses were performed using SPSS software (IBM Corporation, Armonk, NY), version 20.0. For all tests, p≤0.05 was considered to be indicative of statistical significance.

## Results

- Overall characteristics of SGC

**Figure 1:**
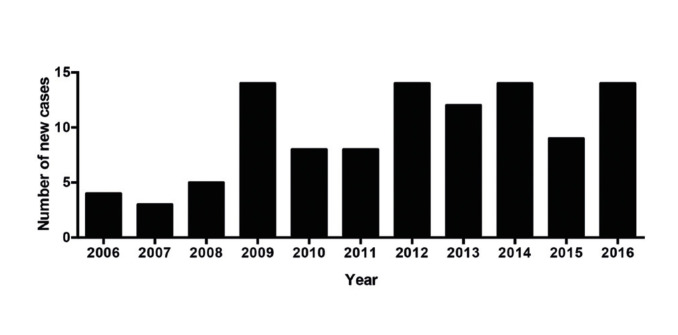
Graph bar of the absolute number of SGC cases diagnosed each year.

One-hundred and seven eligible patients were identified. The number of cases diagnosed each year from 2006 to 2016 is shown in Fig. 1. Overall patient, disease, and treatment characteristics are listed in Table 1. A predominance of female patients was observed, and the mean age at diagnosis was 52 years, ranging from 12 to 93 years. The majority of patients were Caucasian and lived in urban areas. The parotid gland was the most commonly affected site. Most patients reported no symptoms of pain or paresthesia.

**Figure 2:**
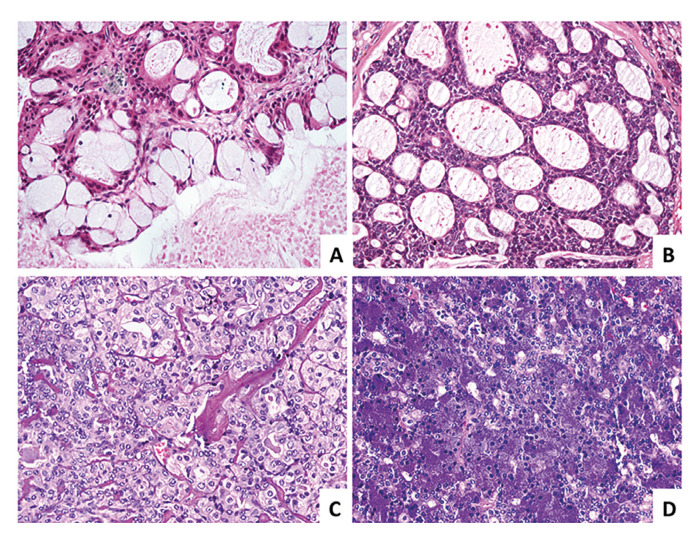
Representative photomicrographs of the most common SGC. (A) Mucoepidermoid Carcinoma. (B) Adenoid Cystic Carcinoma. (C) Carcinoma Ex-Pleomorphic Adenoma. (D) Acinic Cell Caricinoma.

The most common histological types of SGC were mucoepidermoid carcinoma (MEC) (n=39), adenoid cystic carcinoma (AdCC) (n=29), carcinoma ex-pleomorphic adenoma (CExPA) (n=10), and acinic cell carcinoma (AcCC) (n=10). The microscopic aspects of these tumors are illustrated in Fig. 2. Other less common tumors included adenocarcinoma not otherwise specified (n=7), ductal carcinoma (n=4), undifferentiated carcinoma (n=4), epithelial-myoepithelial carcinoma (n=3), and oncocytic carcinoma (n=1).

**Table 1:**
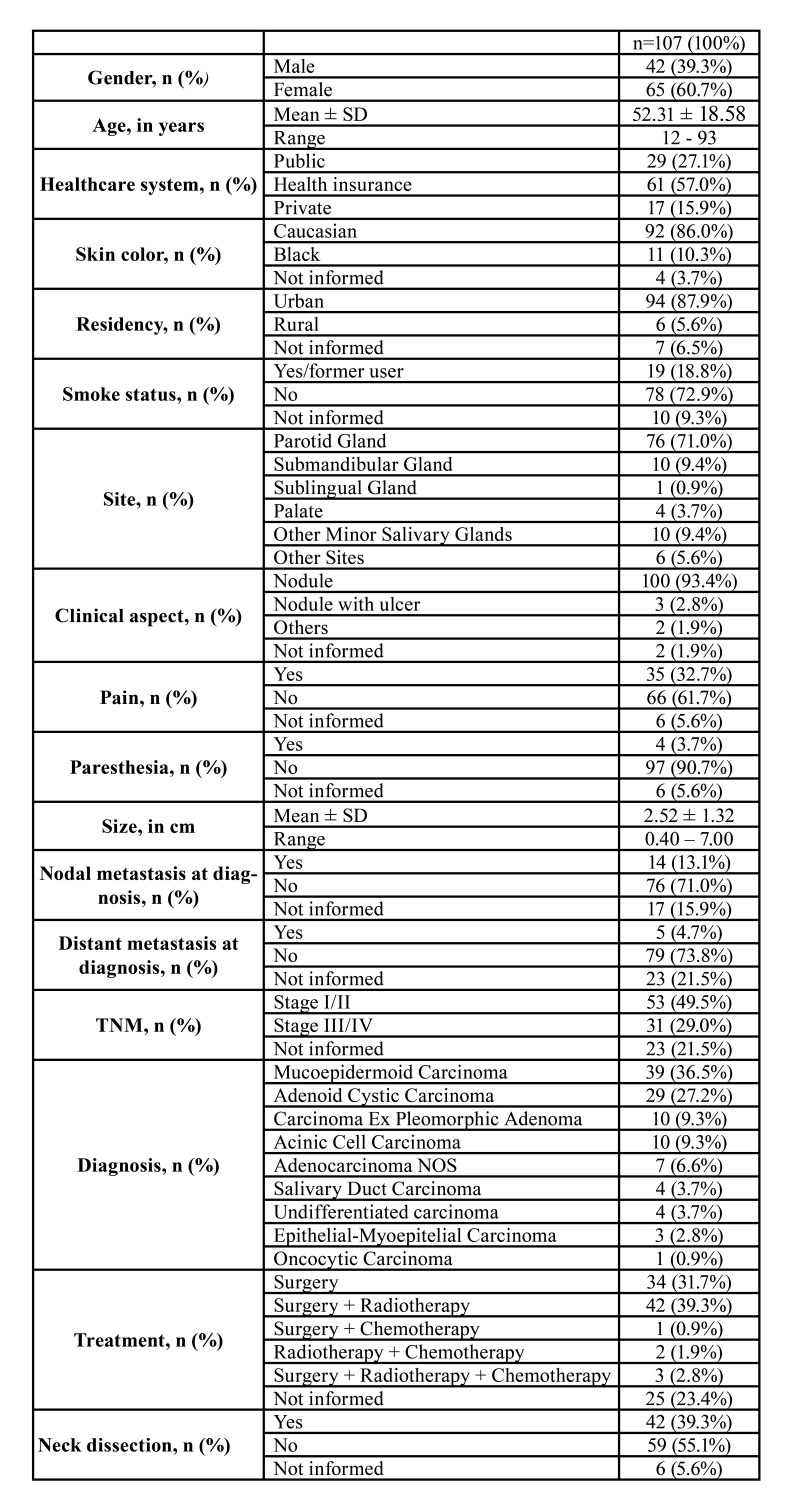
Overall clinico-pathologic and demographic profile of patients diagnosed with SGC.

- Associations of clinicopathological features and SGC type

We compared the clinical and histopathological features with the four most common SGCs: MEC, AdCC, CExPA, and AcCC (Table 2). These histological subtypes had a sufficient sample number to allow a more reliable statistical analysis. Significant differences were encountered for age, pain, and clinical stage. The highest mean age at diagnosis was for patients diagnosed with CExPA (63 years) and the lowest for patients diagnosed with AcCC (43 years) (*p*=0,02). Concerning pain, we observed that whereas the majority of MEC, CExPA, and AcCC cases presented with no pain at diagnosis, half of patients diagnosed with AdCC presented with pain (*p*=0.01). Diagnosis at initial clinical stages (I/II) was most common in MEC, AdCC, and AcCC; however, the majority of patients diagnosed with CExPA presented with advanced clinical stages (III/IV) (*p*=0.04). Although not significant, a tendency of association between type of SGC diagnosis and perineural invasion was also observed (*p*=0.06). In MEC and AcCC, this event was less frequent compared to CExPA and especially to AdCC.

- Associations of MEC grade or AdCC pattern with histological findings

The histological subtype of AdCC and grade of MEC were evaluated through the examination of all slides obtained from the surgical specimen. Among AdCC, 55.2% of cases were classified as cribriform, 27.6% as tubular, and 17.2% as solid. A significant association with AdCC histological subtype and perineural invasion was detected (*p*=0.01 – Chi-squared test). Prevalence of perineural invasion in cribriform, tubular, and solid AdCC was 38.5%, 87.5%, and 0%, respectively. Only two cases of AdCC presented with perivascular invasion (one cribriform and one solid type); thus, no correlation was detected between this feature and histological subtype (*p*=0.33 – Chi-squared test). Concerning MEC diagnoses, 61.5% of cases were classified as low grade, 15.4% as intermediate grade, and 19.9% as high grade. A significant association of MEC grade and perineural invasion was also observed (*p*=0.04 – Chi-squared test). The prevalence of perineural invasion in low, intermediate, and high MEC was 8.3%, 50%, and 33.3%, respectively. Perivascular invasion was observed for only one case of low-grade MEC.

- Survival analysis

**Figure 3:**
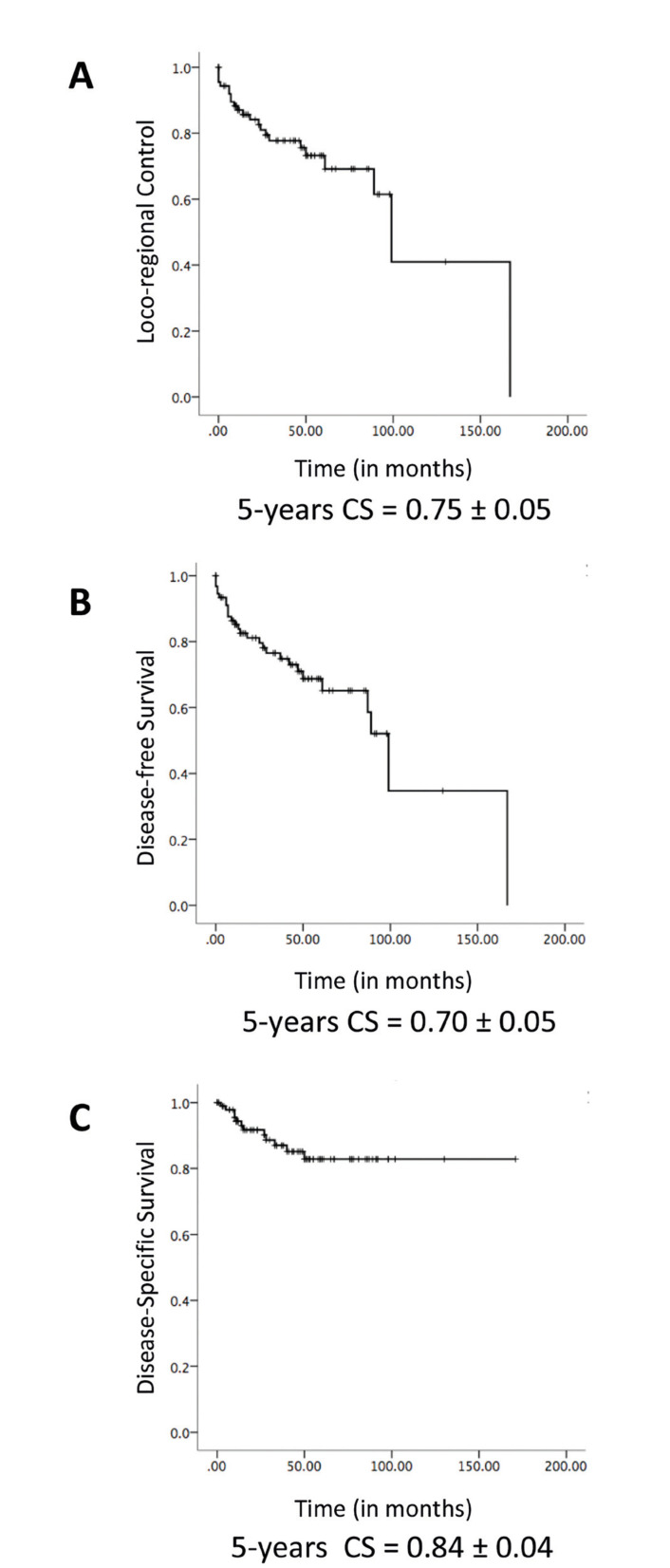
(A) Loco-regional control (LRC) survival curve and 5 year event free cumulative survival (CS) rate ± standard error. (B) Disease-free survival (DFS) survival curve and 5 year event free CS rate ± standard error. (C) Disease specific survival (DSS) survival curve and 5 year event free CS rate ± standard error.

During follow-up, 20 (18.7%) patients presented local recurrence, and 4 (3.7%) patients presented late node metastasis (considered as loco-regional failures). Moreover, 14 (13.1%) patients exhibited distant metastasis, most commonly to the lungs. Combined, these cases were considered as failures in disease-free survival. Eleven (10.3%) patients died due to disease progression during follow-up. Survival curves concerning LRC, DFS, and DSS are presented in Fig. 3.

**Table 2:**
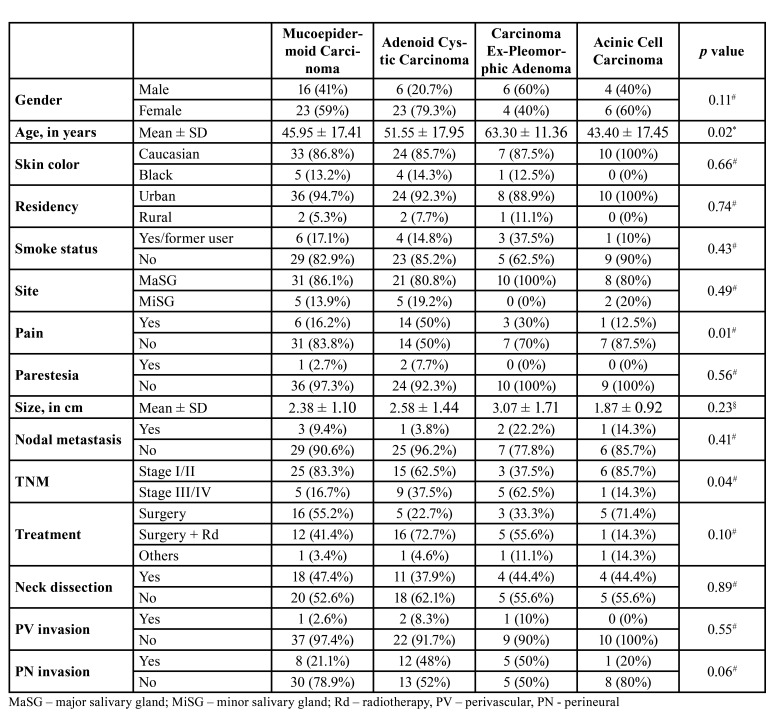
Differences in clinico-pathologic features among most prevalent SGC diagnoses.

Cumulative survival at 5 years for LRC, DFS, and DSS was 75%, 70%, and 84%, respectively.

A univariate Cox regression model was used to determine possible prognostic factors associated with LRC and DFS (Table 3). No model was constructed to DSS due to the low number of events. We observed that the following features were associated with failure in LRC: rural residency (*p*=0.008), presence of nodal metastasis at diagnosis (*p*=0.008), and presence of perineural invasion (*p*=0.02). Moreover, patients diagnosed with MEC had better LRC rates compared to patients diagnosed with AdCC (*p*=0.03). For DFS, the following features were associated with poor prognosis: advanced age (*p*=0.03), rural residency (*p*=0.01), being a smoker or former smoker (*p*=0.01), pain (*p*=0.03), presence of nodal metastasis at diagnosis (*p*<0.001), need for chemotherapy during treatment (*p*=0.02), neck dissection (*p*=0.02), and presence of perineural invasion (*p*=0.01). Similarly, MEC was also associated with a better prognosis regarding DFS compared to AdCC (*p*=0.02). Within MEC and AdCC cases, tumor grade and microscopic subtype, respectively, were not associated with DFS.

**Figure 4:**
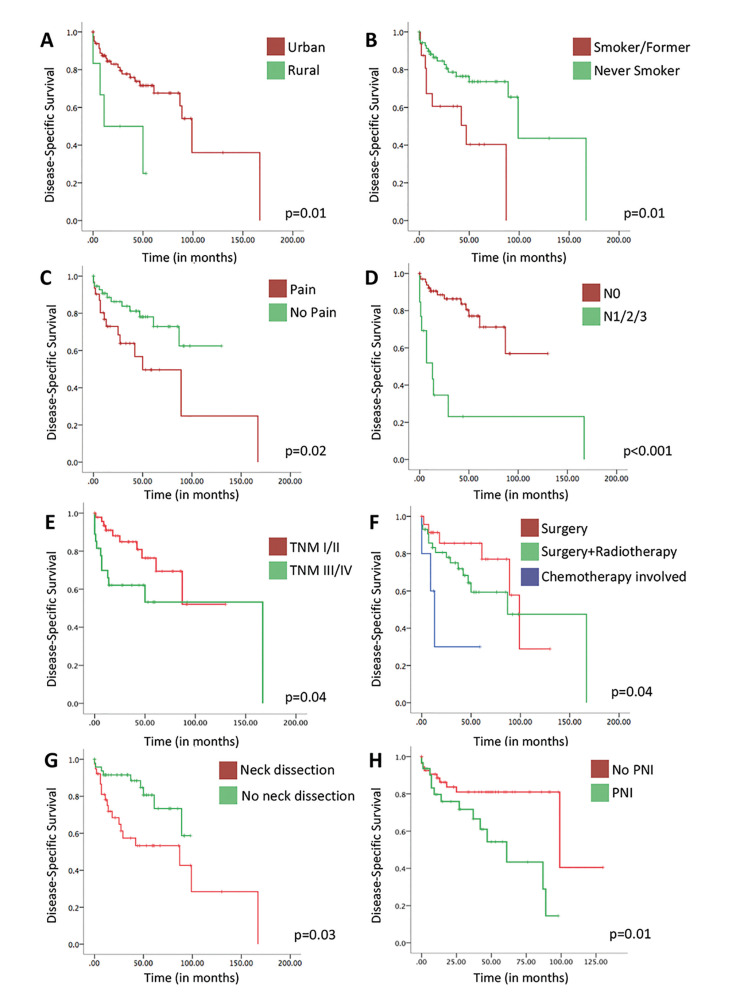
DFS survival curves according to (A) residency, (B) smoke status, (C) pain, (D) nodal metastasis status at diagnosis, (E) AJCC clinical stage (TNM) at diagnosis, (F) treatment, (G) neck dissection and (H) perineural invasion (PNI). Log-rank *p* values are showed for each curve.

The survival curves of prognostic factors of DFS are shown in Fig. 4. In a log-rank test, those prognostic features for DFS remained significant. Moreover, advanced clinical stage (*p*=0.04) was detected as a prognostic factor in this analysis.

**Table 3:**
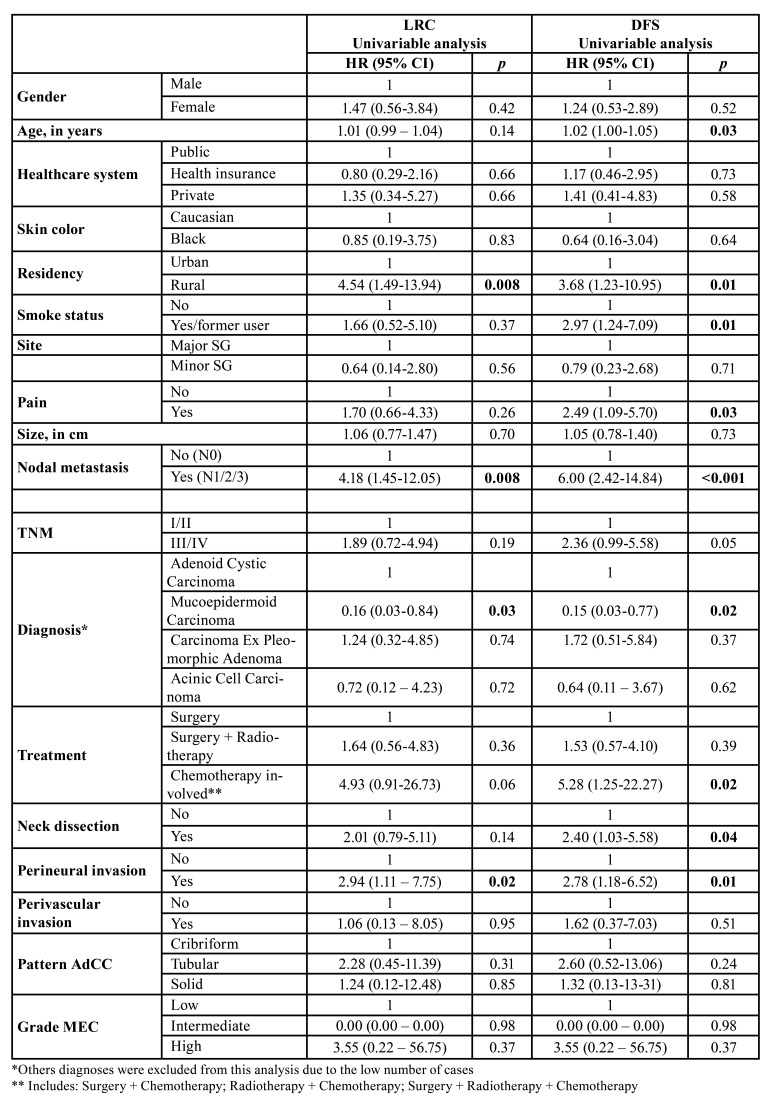
Association between clinico-pathologic features and SGC loco-regional control and disease-free survival estimated by univariate Cox regression.

## Discussion

SGCs are a major challenge for health professionals. As for many rare cancers, insufficient clinical expertise and research interest contributes to significant uncertainties during disease management. In recent years, our group has placed substantial efforts to expand the scientific evidence concerning this type of malignant tumor, through systematic reviews (12), retrospective surveys in reference centers (6,9), immunohistochemical biomarker analysis (13,14), diagnosis based on proteomics (15), and pre-clinical *in vitro* drug testing (16,17). As a group dedicated to contributing to the management of SGC, we detected a lack of recent prognostic studies in an expressive population of Brazil. In 2016, we performed an investigation that included only 24 cases of SGC in Rio Grande do Sul (9). Due to the limited sample size, prognostic markers were not analyzed. Recently, we established an important collaboration with one of the main cancer centers in our state, allowing us to perform a more comprehensive survey. These new data extracted from a representative sample allowed us to compare in a more reliable way the main demographic, clinical, and pathological features of our patients with the world trend. Moreover, we were able to determine the main prognostic markers for SGC in our cohort and also compare with studies from other regions.

The most common SGC type in our cohort was MEC, accounting for 36.5% of all cases. Interestingly, this result is in contrast to what we have observed in 2016, in which AdCC was almost three times more prevalent than MEC among the 24 cases of SGC diagnosed (9). We believe that the present result, of MEC being the most prevalent SGC, is more reliable because the sample is considerably larger and also because it corroborates with other Brazilian surveys with representative SGC samples (5,6,18). Other investigations identified AdCC as the most prevalent SGC, including a study published in 2016 with 871 Danish SGC patients (19) and in 2012 with 282 Finish and 110 Israeli SGC patients (20). Yet, in all these studies, including the present one, a common finding prevails: MEC and AdCC are the two most prevalent SGCs, with a predominance of one or the other depending on geographic region.

In the current study, the male:female ratio was 1:1.5, demonstrating an overall predominance of female patients. This ratio is slightly higher than what was observed by Bello *et al*. (1:1.27) and Bjørnda *et al*. (1:1.05) (19,20). It is now well established that females have an overall slightly increased risk of SGC compared to male individuals. However, this risk appears to be related to histological type, and our results demonstrated that this trend occurs in MEC, AdCC, and AcCC; in contrast, the opposite is noted in CExPA, in which male patients were more frequently affected. A predominance in CExPA male patients was also observed in patients from Helsinki, Finland (20) and in Brazil (7). The risk factors for SGC are poorly understood, and it is not clear whether hormonal changes might play a role in the development of some tumors. However, due to the mean age of patients at diagnosis (around the 4th or 5th decade of life), this hypothesis seems quite unlikely. Yet, it is interesting to note that CExPA (the only tumor with male predominance) was also associated with the highest mean age at diagnosis, in the 6th decade of life. Currently, specific genetic events such as chromosome rearrangements or fusions are being identified as molecular signatures in different types of SGC (21). It is now important to evaluate whether extrinsic or intrinsic factors, such as diet and hormonal changes, can trigger such events.

Site appears to be an important risk factor for SGC, with studies that included both benign and malignant salivary tumors identifying that tumors in minor salivary glands have an increased incidence of malignancy compared to major salivary glands (5,6). Yet, these studies also identified that despite the nature of the neoplasm, major salivary glands are most commonly affected (5,6). In the present work, only malignant tumors were analyzed, and we identified that major salivary glands combined accounted for 81% of cases, with the parotid gland being by far the most affected site. Other studies also conducted in tertiary reference centers have identified a slightly lower percentage of SGC in major salivary glands, such as 63.7% (20), and 55.9% (5).

Age at diagnosis is also considered a risk factor, and most patients are diagnosed in more advanced ages, around the 5th decade of life (22). Our results corroborate with the literature, and we observed that the mean age at diagnosis for SGC was around the 4th and 6th decade of life, with slightly younger patients diagnosed with AcCC and older patients with CExPA. Yet, it is important to highlight that diagnosis at younger ages was identified, with 5 (4.6%) pediatric patients (under 18 years old) and a total of 15 patients (13.8%) under 30 years old. The youngest diagnosis in our study was an AcCC at 12 years old; however, 3 out of 5 (60%) pediatric patients were diagnosed with MEC, being the most common SGC not only in the overall sample but also in younger patients. These results corroborate with Sultan *et al*. 2011 (23) and Cockerill *et al*. 2016 (24) who observed, respectively, a percentage of 49% and 52% of MEC among pediatric SGC patients. Age also represents a prognostic factor. In our study, we found that advanced age at diagnosis increased the risk of local recurrence or disease relapse by 2% for each additional year of life. Hence, a patient diagnosed at age 60 years has a 40% increased risk of relapse compared to a patient diagnosed at 40 years of age. Other studies have found similar results and attributed this fact to probable higher disease stages of older patients and a poorer performance status at the time of diagnosis (19,23). However, it remains unclear whether there are specific age-related differences in SGC biology that could justify a better outcome of younger patients. It is likely that an increased presence of comorbidities in older patients is responsible for hampering the surgical approach or systemic treatment, leading to a lower disease-related survival. Age-related immune system changes may also contribute to worse outcome.

Clinical stage is recognized as an important tool to predict patients’ outcomes and, therefore, is constantly revised to include the most state-of-the-art evidence to determine patients’ classification. In 2017, the AJCC released the 8th edition of head and neck tumors staging system that included some important modifications for oral squamous cell carcinoma (OSCC) and HPV-related oropharyngeal carcinoma; however, no changes were suggested for SGC (10). Our results demonstrated that the majority of patients were diagnosed at initial (I/II) clinical stages (49.5%). Remarkably, CExPA exhibited an inverse pattern of distribution, with 62.5% of patients being diagnosed at higher stages. In the present study, we observed that clinical stage at presentation was significantly correlated with DFS only in the Log-rank test, whereas the Cox regression detected no significant difference. In this analysis, the hazard ratio observed suggested that SGC patients diagnosed in advanced stages would have a 2.36-fold increased chance of presenting disease relapse compared to initial stages; however, the *p-value* was borderline to significance (*p*=0.05). The current staging system seems to be effective in predicting patients’ outcome, although it would be important to understand whether new features could enhance this capacity. One of the new changes incorporated to OSCC in the newest AJCC staging system is the inclusion of pathological analysis of tumor size (pT). The inclusion of depth of invasion in OSCC was justified by the fact that this analysis better discriminates the higher risk of small cancers from those with less invasive capacity, in spite of tumor radial clinical size (10). In SGC, the presence of PNI has been consistently found as a predictor of poor overall survival. We detected that tumors with PNI had 2.78-fold increased chance of presenting disease relapse compared to those tumors without PNI (*p*=0.01), achieving a result with increased magnitude and significance compared to clinical stage. Other studies have found a similar prognostic value for PNI in different samples of SGC (25,26). It would be interesting to test in a more representative sample that allows multivariate survival analysis of whether including PNI as a cut-off event in pT analysis could enhance the prognostic value of the system. Besides age, clinical stage, and PNI (which were already discussed), we identified that smoking status, pain, presence of nodal metastasis, and type of histological tumor as features that could be evaluated at the moment of diagnosis and would indicate higher chances of poor overall survival. Previous studies have been able to determine a significant association between tumor grade and microscopic subtype with MEC (27) and AdCC (28) prognosis, respectively. Despite no significant results observed herein, we believe morphological analysis is of paramount importance. Clinicians must be aware of these factors and establish strategies to overcome disease relapse, such as inclusion of adjuvant treatments or closer follow-up.

Conclusion

The profile of SGC patients observed in the present study corroborated with the most common distribution pattern for SGC described in Brazil and worldwide, concerning age, gender, site, and most prevalent histological types. We identified prognostic factors that can significantly indicate patients at increased risk of recurrence and those who would benefit from closer follow-up. Moreover, some histological features, such as PNI, might deserve further evaluation in larger studies to identify whether this event could enhance the prognostic value of the current staging system.
